# Polycyclic aromatic hydrocarbon in community drinking water, Nsisioken, Nigeria: Source and health risk assessment

**DOI:** 10.5620/eaht.2024015

**Published:** 2024-06-07

**Authors:** Chinemerem C. Nwaozuzu, Stephen O. Abah, Kingsley C. Patrick-Iwuanyanwu

**Affiliations:** 1 Africa Center of Excellence in Public Health and Toxicological Research, University of Port Harcourt, Port Harcourt, River State, Nigeria; 2 Department of Community Medicine, Federal University of Health Sciences, Otukpo, Benue State, Nigeria; 3 Department of Biochemistry, University of Port Harcourt, Port Harcourt, River State, Nigeria

**Keywords:** PAHs, drinking water, UNEP, Nsisioken-Agbi Ogale, Ogoniland, Health risk

## Abstract

In 2011, the United Nations Environment Programme discovered high levels of hydrocarbon pollution in drinking water wells in Nsisioken Agbi Ogale Rivers State, Nigeria. However, the level of polycyclic aromatic hydrocarbons (PAHs) in the same community's water supply was unknown. A comprehensive study of PAHs in three household dug wells and three boreholes was conducted using Agilent 7890B gas chromatography and 5975A mass spectrometry. The detected PAHs were mainly 4 - 5 ringed PAHs, such as Chrysene, Fluoranthene, Pyrene, Benzo[a]anthracene, and Benzo[b]fluoranthene. The total mean concentration was 5.8 ± 2.3 μg/L, with values ranging from not detected in borehole 3 to 8.0 μg/L at well 2. Source identification analysis suggested that the PAHs originated from fuel and biomass combustion. The incremental lifetime cancer risk for children and adults due to groundwater ingestion and skin contact ranged from ND to 7.448 × 10^−3^ and ND to 1.83 × 10^−3^ respectively. The Risk index (RI) values from ingestion and dermal routes were 1.5 ×10^-2^ and 2.4 × 10^-2^, indicating high risk of cancer. The hazard quotient for the two non-carcinogenic PAHs was greater than 1, indicating high toxicity. The PAH concentrations exceeded the maximum contamination limits set by the World Health Organization and the U.S. environmental Protection Agency, highlighting potential health risks associated with water use in the community. Authorities should provide a safe alternative water source for the community.

## Introduction

Approximately one-third of the world's population relies directly on groundwater for drinking, and 40 % of food production depends on groundwater irrigation [1]. In many developing countries, groundwater from boreholes and wells is the most important and reliable resource to meet water needs, highlighting its critical role [2]. However, groundwater is vulnerable to contamination from a range of natural and anthropogenic sources, including oil spills, agricultural runoff, industrial pollutants, and inadequate sanitation practices [3-4]. In Nigeria, the Niger Delta, an oil-rich region, is facing significant petroleum pollution due to oil spills occurring in the past during the exploration and exploitation of crude oil [5]. These spills are due to leaks in pipelines, wellheads and flow stations, as well as transportation of stolen oil, illegal well drilling, sabotage of petroleum facilities, illegal oil bunkering, artisanal refining and substandard artisanal refining [5]. Therefore, the Nigerian government commissioned the United Nations Environment Program (UNEP) to conduct an environmental study of the Ogoniland region in the Niger Delta. A significant study by the United Nations Environment Programme (UNEP) in 2011 sampled household wells in Ogoniland, particularly in Nsisioken-Agbi Ogale, an Eleme local government area, revealing considerable levels of petroleum hydrocarbons, notably benzene, a known carcinogen, at concentrations 900 times higher than the USEPA’s and WHO’s maximum contamination level for drinking water [6]. At the end of their assessment, UNEP, among other recommendations, recommended follow-up monitoring of the communities in the affected areas.

Polycyclic aromatic hydrocarbons (PAHs) are significant organic pollutants owing to their widespread presence in the environment from both natural and anthropogenic sources [7-8]. The major sources include oil spills, agricultural runoff, domestic and industrial effluents, and activities related to the petroleum industry [9-10]. Additionally, PAHs result from the incomplete combustion of various organic materials, such as coal, oil, gas, plastics, and tobacco, as well as from industrial and automobile emissions [9, 11]. The United States Environmental Protection Agency (USEPA) identifies 16 PAHs as priority contaminants, seven of which are categorized as probable human carcinogens namely Benzo[a]pyrene, Benz[a]anthracene, Benzo[b]fluoranthene, Benzo[k]fluoranthene, Chrysene, Dibenz[a,h]anthracene, and Indeno[1,2,3-cd]pyrene [12,13]. Given the daily necessity of water consumption, exposure to PAHs in drinking water has a severe negative health impact, which includes both short-term and long-term adverse effects on human health, including cardiovascular and respiratory conditions [14], reproductive effects, endocrine-disrupting capabilities [15-16], cognitive functions [17], and various cancers such as lung, skin, and bladder cancers [18].

Furthermore, high levels of PAHs induce oxidative stress and immunosuppressive effects during metabolism [19]. Given their carcinogenic, teratogenic, mutagenic, and toxic properties, understanding the occurrence and distribution of PAHs is key to protecting the health of affected communities. Recent investigations in different parts of the Niger Delta region have demonstratedthe presence of PAHs in different environmental media, such as sediments, groundwater, soils,and edible aquatic species. [20-21]. Health risk assessments from previous studies reported varying levels of cancer risks via dermal and ingestion pathways of exposure [22]. Studies have assessed PAH levels in groundwater within the Niger Delta [23-25]. However, there have been little or no available literature on the investigation of PAHs contamination, source identification, and health risks in household wells in Nsisioken-Agbi Ogale, as the occurrence of PAHs was not investigated during the UNEP assessment. Thus, it is still not known the contamination level and health risks.

Therefore, this study aimed to investigate the occurrence of PAHs, source identification, and human health risks associated with PAHs in household wells in the Nsisioken-Agbi Ogale community of Ogoniland, Nigeria. The study represents the first attempt to comply with UNEP recommendations to monitor drinking water quality in all household and public drinking water wells within the spill location [6] using sensitive technology such as gas chromatography-mass spectrometry. It provides new insight into the potential health risks posed by carcinogenic compounds in drinking water, underscoring the criticality of ensuring safe drinking water sources, especially in regions susceptible to PAH contamination, in safeguarding public health and advancing our understanding of the impact of environmental pollutants on water sources.

Specifically focusing on Nsisioken-Agbi Ogale community, this study is motivated by the alarming concentration of benzene as high as 9280 μgL-1 found in a drinking water well by UNEP, greatly surpassing USEPA reference limits of 0.2 μgL-1 [26]. Thus, understanding the occurrence of PAHs and their levels is key to uncovering the source of PAHs, health risks, and finding ways to mitigate exposure. Reassessment of groundwater after a considerable period of time allows us to understand the long-term effects of PAH contamination, potential changes in contamination levels, and the extent of spread. Additionally, new sources of contamination may have emerged, warranting timely intervention to safeguard public health. By assessing health risks and identifying the sources of PAHs, regulatory actions can be informed thereby ensuring community access to safe drinking water.

## Methods

### Brief description of the Niger delta region

**Figure 1.**
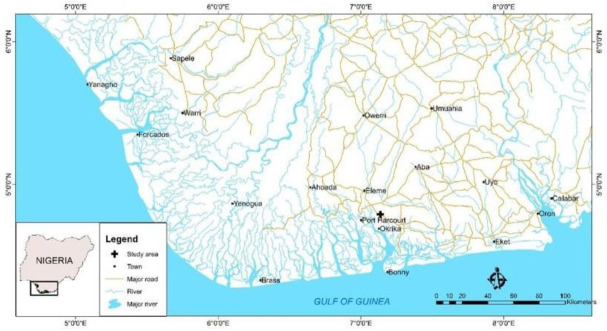
Regional Map of Niger Delta showing study area and major cities Source Edet et al., 2011

The Niger Delta region is the oil-rich region of Nigeria and has been the center of oil exploration since the 1950s. It lies between 50 19' 20.40" N and 60 28' 8.99" E (Figure 1). The Niger Delta Basin occupies the continental margin of the Gulf of Guinea in equatorial West Africa. [27-28], has a total area of about 75,000 km2 and occupies the coast and part of the ocean of the Benue Trough, which accounts for 7.5% of Nigeria's landmass [29]. Over the past 55 years, contamination of soil and water resources by petroleum hydrocarbons has become a serious environmental problem and a risk to human health due to the carcinogenic and mutagenic properties of some of the various hydrocarbon compounds [30].[Fig f2-eaht-39-2-e2024015]

### Description of sampling locations

Ogoniland is a region in the Niger Delta that lies east of Port Harcourt in Rivers State, Nigeria, close to the Gulf of Guinea coast. It comprises four Local Government Areas (LGAs) of Khana, Gokana, Tai, and Eleme. This study was conducted in Nsisioken-Agbi Ogale, in Eleme Local Government Area. The capital city of Port Harcourt is roughly 40 kilometers away from Ogale, which is situated between latitudes 40 47'13.6" N and longitudes 70 7' 36.6 E. It is bounded by Ogale, Okuluebu, Akpangbala, and Nchia communities. The study area was chosen on purpose. Purposive selection was made because UNEP investigated several hand-dug wells and boreholes in the community and found that the concentration of bezene in drinking water wells was 900 times higher than the WHO reference level [6].

Rainfall in the study area is seasonal, variable, and heavy, occurring mainly from May to October while the hottest moths are February through May. The average annual rainfall of about 2,400 mm and an average annual temperature of about 27 °C. The local residents are predominantly farmers, fishermen, and traders, while the company workers, civil servants or government employees reside mostly in the urban centers. Table 1 shows the description of the sampling locations as well as the water usage.

### Water sampling and in-situ analysis

Groundwater sampling took place between April 2022 and February2023 in the morning hours between 10:30 a.m. and 12:00 p.m. Water samples were collected in duplicate using pre-washed 400 mL glass bottles from six different sampling sites or households, namely: three excavated wells labeled W1, W2 and W3 and three boreholes labeled BH1, BH2 and BH3. A total of twelve water samples were taken. At each collection point, the bottle was first rinsed with the water to be examined. The samples were then labeled accordingly, indicating the location of sampling, the date and time of sampling, and the unique sample ID. The sample was stored in a cool box with ice blocks to prevent possible microbial decomposition of PAHs or photolysis by UV rays prior to transportation to the laboratory for analysis. Other reasons for storage in a cool box were to preserve the integrity of the samples such as minimizing loss due to the volatile nature of some PAHs and reducing chemical reactions that can alter the concentration of PAHs if exposed to high temperatures [31-32].In the laboratory, the samples were stored in the refrigerator before analysis. Physical parameters such as color and smell were observed and documented in the field notebook. Other parameters such as pH, temperature, electrical conductivity (EC), total dissolved solids (TDS), dissolved oxygen and turbidity were determined using the Hanna Multiparameter Meter HI9828. In the laboratory, the samples were stored in the refrigerator before analysis.

### Sample preparation, reference standards, and PAH extraction

All the reagents used in this study were of high-purity analytical grade. The water was examined for the presence of the 16 priority PAHs listed by the USEPA. The PAHs include naphthalene (Nap), acenaphthylene (Acy), acenaphthene (Ace), fluorene (Flu), phenanthrene (Phen), anthracene (Ant), fluoranthene (Fla), pyrene (Pyr), benzo [a]anthracene (BaA), chrysene (Chr), benzo[b]fluoranthene (BbF), benzo [k]fluoranthene (BkF), benzo [a]pyrene (BaP), indeno [1,2,3-cd] pyrene (InP), dibenzo [a,h] anthracene (DbA), and benzo [ghi]perylene (BghiP). A PAH standard mix solution of 16 United States Environmental Protection Agency (USEPA) priority PAHs, each at 1000 μg/L in dichloromethane, purchased from Accustandard, USA, was used as the standard. Gas chromatography/mass spectrometer (GC/MS)-grade dichloromethane (DCM) and anhydrous sodium sulfate were purchased from Accustandard Inc., USA. Five deuterated PAHs, including naphthalene-d8, acenaphthene-d10, phenanthrene-d10, chrysene-d12, and perylene-d12, were selected as internal standards (IS) and were all purchased from AccuStandard Inc., USA.

PAHs in water were extracted using liquid-liquid extraction. The extraction procedure was carried out in accordance with an established procedure previously described in a study [33]. For liquid-liquid extraction, dichloromethane (DCM) was used three times in a volume of 50, 25, and 25 mL. A 400 mL water sample was poured into a separation flask and mounted on a retort stand. The samples were mixed with 1μL of a 100μg/L standard mixture consisting of 16 PAHs. To extract PAHs, 50 mL of dichloromethane (DCM) as extraction solvent was added to the flask, which was shaken vigorously and depressurized at regular intervals. The material was allowed to stand until two distinct layers had formed in the flask. The bottom layer, the organic extract, was collected in a clean beaker. The process was repeated twice: returning the aqueous layer to the separatory funnel, adding another 25 ml of DCM and shaking vigorously for 1 min with periodic aeration. Here too, two layers of the sample were allowed to separate and the bottom layer was collected in a glass collecting flask. The three parts of the organic extract were combined and dried by adding2 g of anhydrous sulfate. The dried extract was then further concentrated using a rotary evaporator (9 Hei-VAP Core hand-lift model with G1 diagonal glass instruments) to a final volume of 1μL, which was transferred to GC vials and subjected to gas chromatography-mass spectrometry (GC-MS). Analysis.

### Gas chromatography analysis and condition

The GC-MS detector used was an Agilent 7890 GC instrument (Agilent Technologies, Santa Clara, CA, USA) coupled with a 5975 mass spectrometer detector (MSD). (Agilent Technologies Inc., California, USA) A DB-5MS capillary column (30 m × 0.25 mm i.d. × 0.25 μm, J&W Scientific, USA) and an Agilent 7683B autosampler were used to inject the sample, and the split opened after 1 min to drain the solvent. The inlet temperature was set at 290 °C and the inlet volume was 1μL in spitless mode. The initial GC oven temperature was 65 °C, held for 1 minute, then increased to 140 °C at 25 °C/minute (Ramp 1), and then increased to 290 °C at 10 °C /minute for 11 minutes (Ramp 2) Helium was used as the carrier gas with a flow rate of approximately 1 mL/min, an initial pressure of 9.0855 psi, and an average velocity of 37.604 cm/s. The mass spectrometer used an electron ionization energy of 70 eV. The following operating conditions were used: source temperature of 230 °C; quadrupole temperature of 150 °C; multiplier voltage of 2000 V; Interface temperature of 310 °C. The instrument was equipped with an electron impact ion source (EI) and operated in Selective Ion Monitoring (SIM) mode to monitor the ions of the target PAHs.

### Quality Control/Assurance

#### Calibration and method validation

The stock solution of each PAH (1000 μg/mL) was diluted to obtain a working standard solution, which was further serially diluted to prepare seven calibration standards with concentrations ranging from 0.05 to 7.0 μg/L. The samples were spiked with 1 μL of a 100 μg/L standard mixture consisting of 16 PAHs, which was used as an external standard to calibrate the PAHs. The PAHs in the water samples were quantified using an external standard. The concentration-response plot was used to construct the calibration curve for quantification purposes and to define the limit of detection (LOD) and acceptable correlation coefficient (r = 0.99). Individual PAHs have limits of detection (LOD) and limits of quantification (LOQ) ranging from 0.02 µ g/l to 5.0 µ g/l. Before extraction, four surrogate standards were added to the sample to monitor the recovery of individual PAH compounds. Substitute standards used include acenaphtene-d10, chrysene-d12, phenanthrene-d10 and perylene-d12. The samples were subjected to the same extraction procedure as described above. The following equation was used to calculate replacement recovery percentage.


(1) %R = Qd/Qa x 100

where Qd is the quantity determined by analysis and Qa is the quantity added. For surrogate percent recovery to be acceptable, it must fall within 80–120% [34]. The percentage recovery of PAHs in water is between approximately 75.5 % and 100 %. Compounds were recognized based on their retention times, while quantification was performed by external standardization by the instrument based on the response factor it generated for each congener from the recorded linear curve. [11]. A PAH concentration below the detection limits of the method was classified as undetectable (ND).

### Gas chromatography with flame ionization detection (GC-FID) for TPH analysis

#### Water collection

A total of six water samples were collected from the filed in 400 mL bottle and Samples were preserved in an ice box before taking it to the laboratory refrigerator to be stored at 4 degrees Celsius until their organic extraction. The holding time was 14 days from collection to GC analysis.

#### Sample extraction procedure

The extraction procedure of TPH in groundwater samples using n-pentane solvent extraction was adapted from the procedure by [35] and the groundwater samples were extracted by transferring 10 mL of sample into a 40-mL vial containing 5 g of NaCl. After the addition of 20 mL of n-pentane, the mixture was hand-mixed. The samples were placed in an end-over shaker overnight and centrifuged for 15 min at 300 rpm to facilitate phase separation. Two additional extractions, each with 20 mL n-pentane, were performed in the same manner. 1.0 mL of the organic extract (pentane-extracted layer) was then transferred into a vial using a glass pipette and analyzed by GC/FID.

#### Chromatography conditions

The GC/FID analysis of the TPHs was conducted on a Hewlett Packard 5890 Series II-Plus gas chromatograph equipped with an HP 7673 Autosampler and FID detector, paired with a 30×0.32 mm DB-5 (95 metil-5 %-fenilpolisiloxane) fused silica capillary column. The oven temperature was programmed to increase from 40°C for 3 minutes to 300 °C at a rate of 15 °C per minute. Samples were injected in spitless mode, with the relay open for 20 seconds. The injector temperature was set at 250 °C, while the detector temperature was set at 320 °C. Helium was used as the carrier gas at a linear velocity of 38 cm/sec (15 psig). Data handling was carried out using Agilent Chemstation chromatography software (version 10).

#### Health risk assessment

For human risk assessment, the potential for adverse health effects due to prolonged exposure to carcinogenic and non-carcinogenic compounds was estimated for two groups of populations: adults and children [36]. In this study, previous investigation revealed that the sampled borehole and well water was mainly used for drinking, bathing and washing. On this basis, it was assumed that human exposure occurs through ingestion and through the skin. The human health risk model recommended by USEPA was applied by calculating the potential carcinogenic and non-carcinogenic effects of exposure to PAHs over a period of time. In this study, the average daily dose (ADD), incremental lifetime cancer risk (ILCR), and risk index (RI) were carefully estimated. Health risks from ingestion and dermal contact were calculated using equations 2 and 3.


(2) ADDi = Cw × IR × EF × ED / BW × AT

(3) $${\mathrm{ADD}}_{d}\;=C\;\times \;\mathrm{SA}\;\times \;\mathrm{Kp}\;\times \;\mathrm{ET}\;\times \;\mathrm{EF}\;\times \;\mathrm{ED}\;\times \;\mathrm{CF}\;/\;\mathrm{BW}\;\times \;\mathrm{AT}$$

ADD_i_ is the average daily intake through ingestion (mg/kg day), Cw is the concentration of PAHs in water (μg/L), ADD_d_ is the average daily intake through dermal adsorption (mg/kg day), IRw is the daily water ingestion rate (L/day) (2 L/day for adults and 1 L/day for children), EF is exposure frequency (350-day year-1), ED is exposure duration (26 years for adults and 6 years for children), BW is body weight (70 kg for adults and 15 kg for children), Kp (cm/h) is the dermal permeability coefficient of PAHs (obtained by USEPA, 1993) AT is the average time (non-carcinogens = ED × 365 days), (carcinogen = 70 × 365), SA is skin surface area (19,652 cm2 for adults and 6365 cm2 for children), ET is exposure time for showering and bathing (adults: 0.25 h/day; children: 0.33h/day). The conversion factor (CF) is 0.001 for adults and children [37-40].

The hazard quotient (HQ) for ingestion and dermal exposure was calculated for non-carcinogenic PAHs by dividing the ADD by the reference dose (RfD) for individual contaminants as presented in Eqs. (4) and (5). Hazard index (HI) and the sum of HQs were also estimated for all PAH congeners in the samples using Eq. (6).


(4) $$\mathrm{HQ}\;=\;{\mathrm{ADD}}_{i}\;/\;\mathrm{RfDi}$$

(5) $$\mathrm{HQ}\;=\;{\mathrm{ADD}}_{d}\;/\mathrm{RfDd}$$

(6) HI = ∑ HQs

Furthermore, cancer risk (ILCR) and risk index (RI) were calculated for carcinogenic PAHs detected in the water samples using ADD_i_ and ADD_d_ (mg/kg/day) for ingestion and dermal pathways. In accordance with USEPA recommendations, Eqs. (8-10) were used to calculate the CR and RI in the water sample [37].


(7) ILCR = CDI × Cancer oral slope factor (CSF)

For ingestion


(8) $$\mathrm{CRi}\;=\;{\mathrm{ADD}}_{i}\;\times \;\mathrm{CSF}$$

For dermal contact with water


(9) $$\mathrm{CRd}\;=\;{\mathrm{ADD}}_{d}\;\times \;\mathrm{CSF}$$

(10) RI = ∑ CR

Where CSF is the cancer slope factor for each PAH congener. CSF for benzo(a)pyrene is 0.73 mg/kg/day [38]. The measured carcinogenic PAH concentrations were multiplied by their respective Toxic Equivalent Factor (TEQ) to yield their benzo(a)pyrene-equivalent (BaPeq). Statistical analysis calculating mean and standard deviation were performed using MS Excel office 2021.

## Results

### Physico-chemical properties of groundwater in the study area

Table 2 shows the concentration values of the physical and chemical properties of water samples.

PH: The PH range from 6.54 - 8.2.

Temperature: The mean temperature of the groundwater from wells and boreholes were 29.4 ± 0.17 °C and 29.9 ± 0.06 °C respectively. The values ranged from 29.6 °C in W1 to 30 C in BH2. From the table it can be seen that the borehole waters (BH1 and BH2) have higher temperature.

Electrical conductivity (EC) was higher in well waters, ranged from 11.2 μScm^-1^ in BH3 to 500 μScm^-1^ in W1

Total Dissolved Solute: The TDS in water samples ranged from to 200.9 mg/L to 330.9 mg/L in well waters and from 6.09 mg/L to 20.4 mg/L in boreholes and both are within WHO permissible limit of 500 - 1500 mg/L (Table 2).

Turbidity: The turbidity values ranged from 2.7NTU in BH3 to 9.3 NTU in W1. The mean turbidity for well and borehole waters is 7.6 ± 1.86 NTU and 3.56 ± 1.33 NTU respectively.

DO: The dissolved oxygen for well and borehole is 3.58 ± 0.28 mg/L and 4.59 ± 0.68 mg/L respectively.

Borehole waters have higher dissolved oxygen than the well waters.

### Sulphate, nitrate and phosphate

Sulphate ranges from 0.741 mg/L in W1 to 0.001 mg/L in BH 2. The mean sulphate value for well water and borehole waters are 0.37 ± 0.33 mg/L and 0.05 ± 0.06 mg/L respectively. The mean nitrate is 0.24 ± 0.001 mg/L for well waters and 0.24 ± 0.009 mg/L for borehole waters. The mean phosphate for wells is 0.07 ± 0.03 mg/L and 0.07 ± 0.03 mg/L.

Total Petroleum Hydrocarbon (TPH): The TPH ranged from Not detected in BH3 to 13,751.1 μg/L in BH2. The mean total petroleum hydrocarbons for well water are 3012.1 ± 1786.5 μg/L and 10580.15 ± 4484.33 μg/L.

From Table 2, it is apparent that most of the water parameters such as Electrical conductivity EC, turbidity, and total dissolved solids TDS have higher concentrations in well waters than in borehole waters. Whereas, borehole waters are richer in dissolved oxygen. Among borehole water, BH1 has highest concentrations of total petroleum hydrocarbon.

### PAH concentration in groundwater samples

The concentrations of 16 priority PAHs in groundwater from domestic wells and boreholes in Nisioken-Agbi Ogale, Nigeria are summarized in Table 3. PAHs were detected in all samples except BH3 at concentrations ranging from ND to 2.0 μg/L. Only five of the sixteen PAH pollutants were detected in the test samples, with a mean total concentration of 5.8 ± 2.3 μg/L. Fluoranthene has the highest mean concentration of 8.5 ± 2.13 μg/L, followed by chrysene (7.5 ± 2.12 μg/L), pyrene (7.0 ± 2.84 μg/L) and benz[a]anthracene (5.5 ± 3.54 μg/L), while benzo(b)fluoranthene has the lowest mean concentration (0.5 ± 0.71 μg/L). The mean PAH concentration ranged from N.D. in BH3 to 9.0 μg/L in W3. W1, W2, W3 showed a higher PAH concentration with a ∑PAH concentration of 8.0 μg/L compared to the borehole water, which had a total ∑PAH concentration of 2.5 μg/L. No PAH was detected in BH3.

### PAHs composition

**Figure 3.**
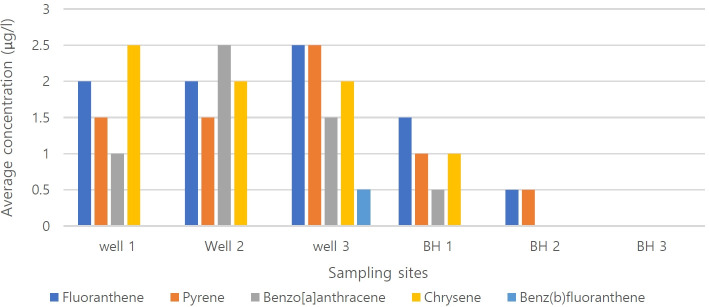
Profile of individual PAHs found in household wells and boreholes in Nsisioken-Agbi Ogale, Nigeria..

The composition diagrams of PAHs in Nsisioken-Agbi groundwater are shown in Figure 3. Fourto Fiveringed PAHs (fluoranthene, pyrene, chrysene, benz[a]anthracene and benzo[b]fluoranthene) were predominant in water samples and contained 75 %, 75 %, 50 %, 58.3 % and 8.3 % detection of frequency of the total PAHs respectively. The PAHs detected in the highest concentrations are fluoranthene and chrysene. In general, 4-ring PAHs dominated in water samples. Fluoranthene and pyrene were present in all. From these data, BH 1, which serves as a drinking water source, yielded the lowest PAH and TPH levels, while no levels were detected in BH 3, an interventional water project.

### PAH source identification using the diagnostic ratio

The main sources of PAH contamination are petrogenic (oil spills, oil exploration), pyrogenic (burning of fossil fuels and other organic matter), and diagenetic sources (decomposition and transformation of natural organic matter) [46]. PAH concentration ratios such as the ratio of benz[a]anthracene (BaA) to the sum of benz[a]anthracene (BaA) and chrysene (Chry), represented by, BaA/(BaA + Chry) and ratio of fluoranthene (FLA) to the sum of fluoranthene (FLA) and pyrene (Pyr) represented as FLA/(FLA + PYR) are used in environmental samples as unique chemical tracers to identify possible sources of PAHs. Others include: the ratio of phenanthrene/anthracene, (PHEN/ANT), Anthracene and the sum of Anthracene and Phenanthrene (ANT/ANT + PHEN), indeno [1,2,3-cd] pyrene and the sum of indeno [1,2,3-cd]pyrene and benzo[g,h,i]pyrene, IcdP/(IcdP + BghiP) [47]. In this study, PAH sources in water samples are determined using the following isomer ratios: Fla/(Fla+Pyr) and BaA/(BaA+Chry, as shown in Table 4.

To identify the potential sources of contamination in this study, the ratios Fla / (Fla + Pyr) and BaA / (BaA + Chry) were used. The ratio Fla / (Fla + Pyr) > 0.4 - 0.5 indicates fuel combustion. The ratio Fla / (Fla + Pyr) > 0.5 indicates a pyrolytic source. [44]. Ratios of BaA / (BaA + Chry) indicated a petrogenic origin at a ratio < 0.2, ratios > 0.35 indicated pyrogenic sources, [44] and ratios between 0.2 and 0.35 indicated a mixed source [45]. In this study, the ratio of BaA / (BaA + Chry) > 0.3 showed fuel and biomass combustion, suggesting that the PAHs are mainly of pyrolytic origin (Table 5). In general, the diagnostic ratio should suggest that the water PAHs come from fuel and biomass combustion (burning coal, wood, grass). Both diagnostic ratios indicated that PAH sources in water samples came from petroleum and biomass combustion.[Fig f4-eaht-39-2-e2024015]

### Health risk assessment

#### Non-carcinogenic risk assessment

Two routes of exposure to water were considered: dermal and ingestion. The risk was calculated for adults and children. The hazard quotient is typically calculated for non-carcinogenic substances to determine the health concerns of PAHs. In order for there to be a significant risk of non-carcinogenic effects from the individual PAHs, the HQ should be greater than one for all PAHs in total [49]. Table 6 and 7 (see Supplementary Materials) show hazard Quotients (HQs) of PAHs in the water samples by Ingestion and dermal contact.

#### Carcinogenic risk assessment

Tables 8 and 9 (See Supplementary Materials) show the life cancer risk of PAHs from ingestion and dermal exposure. Of the five PAHs found, three (BaA, Chry and BbF) are classified as carcinogenic to humans [12]. The health risk due to exposure to them was assessed. The ILCR was calculated for exposure to PAHs in children and adults. The results show that the ILCR for three carcinogenic PAHs in water samples from the ingestion exposure route ranged from ND to 7.44 × 10^-3^ for children and ND to 1.83 × 10^-3^ for adults. At dermal exposure, the ILCR for three carcinogenic PAHs in water samples ranged from ND to 1.7 × 10^-3^ for children and ND to 3.69 × 10^-3^ for adults. Tables 8 and 9 summarize the RI (sum of ILCR) for each of the three carcinogenic PAHs from each of the sampling locations. According to the regulatory guidelines, ILCR ≤ 10^−6^ indicates no or negligible risk. ILCR greater than 10^-4^ denotes a high risk of harmful health outcomes, like cancer. The risk is considered acceptable between two extreme ILCRs. [50]. The ILCRs calculated in this study are not within the acceptable limit. The Risk index (RI) values from ingestion and dermal pathways are 1.5 × 10^-2^ and 2.4 × 10^-2^

Overall, these results indicate that groundwater in Nsisioken-Agbi Ogale in Eleme, Nigeria is contaminated by HMW-PAHs and PAHS are above WHO acceptable limits. Borehole 2, which serves as a drinking water source for the community, has a high non-carcinogenic risk. This poses a potential health risk to community members who consume this water. The cancer risk scores of BaA and BbF indicated a higher risk for children and adults in W1, W2, W3 and BH1, while BH2 showed no cancer risk.

## Discussion

### Physico-chemical characteristics of groundwater

This study examined the presence of PAHs in drinking water wells in Nsisioken-Agbi, their potential source, and carcinogenic and non-carcinogenic health risks a decade after the UNEP study. The aim was to ensure the safety of drinking water in the community. This research, in line with the UNEP recommendation, set out with the aim to is to reassess water supplies in oil-affected communities, particularly Nisioken-Agbi for PAHs contamination, source, and health risks.

### PH, temperature, and electrical conductivity (EC)

The pH value in all samples falls within the maximum WHO permissible limit of 6.5–8.5 for drinking water. The temperature in all well water samples ranged from 29 °C to 29.9 °C. A study reported similar results (29 °C) for well water in some villages in Nigeria [51]. Temperatures in BH1 and BH2 (30 °C) are slightly higher than the other samples. This can be attributed to the ability of plastic water storage tanks exposed to sunlight to absorb heat. The plastic material acts as an insulator, trapping heat and warming the water inside. Electrical conductivity is higher in well water (500 μS/cm, 310.9 μS/cm, and 385.5 μS/cm) than in boreholes (32.78 μS/cm, 12.84 μS/cm, and 11.21 μS/cm). All EC values are within the WHO permissible limit. According to the EPA, EC should not exceed 1000 μS/cm [43].

### Dissolved oxygen, total dissolved solids, turbidity

On the other hand, high dissolved oxygen levels improve the taste of drinking water. The higher the DO levels, the better the water quality. DO levels in all water samples were below the permissible limit of 6.5 - 8 mg/L [42]. DO levels in this study are higher in boreholes than in wells, suggesting better water quality in the boreholes. Total Dissolved Solids refers to the total concentration of dissolved substances. Water containing more than 500 mg/L of TDS is not considered fit for drinking water supplies. The mean TDS value of the samples is 261.3 mg/L, ranging from 330.9 mg/L in well 1 to 6.09 mg/L at BH3. The TDS values of all the water are lower than the 500 mg/l limit prescribed by the WHO. Waters sampled from W1, W2, and W3 exhibit higher turbidity values as they lack relative clarity and appear a bit cloudy. Turbidity results from various materials suspended in water, e.g., clay, silt, organic matter, algae, and microscopic organisms. Among the boreholes, only BH1 exceeded the WHO limit of 5 NTU.

### Sulphate, Nitrates, Phosphate

The sulfate concentration in this study ranged from <0.001 to 0.74 mg/L, well below the permissible limit of 250 mg/L [43]. Sulfur above the recommended level may lead to an unpleasant taste and odour in water. The mean nitrate levels in the current investigation are 0.24 mg/l, which is well below the WHO safety limit [44]. There is no specific WHO guideline for phosphate in drinking water, however, maintaining low levels is essential to maintaining water clarity. Sulfate, nitrate, and phosphate in waters from the study area are all within the WHO permissible limit and may not be harmful to humans. The analysis showed that some of the physicochemical properties of the water samples were within the WHO and USEPA permissible values. A disturbing finding is the total petroleum hydrocarbon (TPH) content, which in all water samples significantly exceeded the WHO and Occupational Safety and Health Administration permissible limits of 500 mg/L [45]. However, most of the physical parameters of the water in this study are consistent with data obtained by another study [52], which assessed the groundwater and soils around the Afam area of Port Harcourt in the Niger Delta, where most of the physical and chemical parameters measured in the sampled groundwater were below WHO reference values.

### PAH concentration

The presence of PAHs in our study samples indicates persistent PAH contamination in the environment, even years after the UNEP study. None of the water samples showed physical signs of contamination, such as oil films or the smell of crude oil. However, the water from BH1 had a strong odour and a higher concentration of PAHs compared to BH2. A plausible explanation could be the proximity to a local landfill. A study found that municipal waste from industrial sites and trash contributed to increased concentrations of PAHs in soil at landfills, which can subsequently leach into groundwater [53]. Notably, BH1 was reportedly only used by residents for bathing and laundry, while BH2, despite containing PAHs, was used by many for drinking.

Furthermore, BH1 had the highest TPH concentration. In contrast, PAHs and TPH were not detected in BH3, an intervention water source provided by the Rivers State Government to mitigate groundwater pollution in Ogoniland, in line with UNEP recommendations. Upon investigation, it was found that BH3's water came from a neighboring community, Alesa, which is free of oil pollution. The overall mean PAH concentration in our study was comparable to that reported by Edet et al. who examined PAHs in groundwater and soils near an oil spill site in Eleme [25],but was lower than that reported by [23-24]. All PAH concentrations exceeded the USEPA recommended maximum contamination level (MCL) of 0.2 μg/L for drinking water, consistent with similar studies in the Niger Delta.

### PAH composition and source Identification

It is somewhat surprising that petrogenic PAHs, that is PAHs originating from crude oil and petroleum sources was not found. Instead, the composition of PAHs showed a dominance of 4- and 5-ring HMW-PAHs and the absence of LMW-PAHs such as 2-3-ring PAHs. The dominant HMW-PAHs in this study is consistent with the findings of a study assessing drinking water in Tehran, Iran [54]. However, our results do not support previous studies in some parts of the Niger Delta, where 2-3 ring PAHs were predominant in groundwater [23-24]. This unexpected finding could be attributed to the volatilization of light PAHs due to their relatively high vapor pressure, making them undetectable. On the other hand, since HMW-PAHs are more stable, they can persist in the environment by adsorbing soil particles. The higher concentration of HMW-PAHs, especially in hand-dug wells, may be due to their ability to adsorb solid particles and persist longer in the environment. Although HMW-PAHs have low water solubility, their adsorption on suspended dust can lead to contamination. PAHs in the environment typically stem from petrogenic, pyrogenic, or biogenic sources. Identifying these sources is crucial for developing effective pollution control strategies. This current study used diagnostic ratios to analyze possible PAH sources in the study samples. The results suggested that the PAHs in this study originated from fuel and biomass (coal, wood, grass) combustion.

### Human health risk assessment

The non-carcinogenic risk analysis in this study found that the water sources in the community were not safe for consumption because they posed systemic health risks with a hazard quotient greater than 1. Children have been found to be at higher risk than adults. The incremental lifetime cancer risk (ILCR) from both ingestion and skin exposure routes exceeded WHO and USEPA permissible limits. These results are consistent with a systematic review by Ofori et al. [22] which highlighted low-to-high cancer risks from PAH exposure in the Niger Delta.

One of the strengths of our study is that the data collection process was thorough and the water samples were analyzed using GCMS with a lower limit of detection, which provided more accurate, precise and reliable measurements than GC-FID. The study shows that the occurrence of PAHs has not been eliminated over time. The implication is that the consumers of these water stand a high risk of health effects hence, alternative, safe water or intervention water in which no PAHs or TPH have been found should be made the community's main water source. This is particularly a useful finding.

## Conclusions

The main goal of this current research was to assess contamination of groundwater by polycyclic aromatic hydrocarbons (PAHs) in Nsisioken-Agbi Ogale, a Niger Delta community affected by historic oil spills, identify source and assess health risks associated with consuming the waters. The study has identified PAH contamination, primarily with high molecular weight (HMW) PAHs, which indicates a pyrogenic source, likely derived from fuel and biomass combustion. Alarmingly, the water was found to be unfit for consumption due to its high hazard quotient and cancer risk, particularly to children. This finding highlights the urgent need for restoration efforts and the provision of safe drinking water sources to protect public health. This finding will be of interest to stakeholders and policy makers as it draws their attention to the fact that safe drinking water is a serious challenge for the Nsisioken-Agbi Ogale community in Nigeria. These findings provide information on urgently needed measures for clean drinking water in the affected community.

This study is limited by a small sample size because it focused on a specific community. The study was also limited by financial constraints and the expensive nature of water analysis using GCMS. Notwithstanding these limitations, the study produced crucial finding and crucial insights for safeguarding public health. Further research on the current topic which include more sites and larger samples is therefore recommended to help establish this finding.

## Figures and Tables

**Figure 2. f2-eaht-39-2-e2024015:**
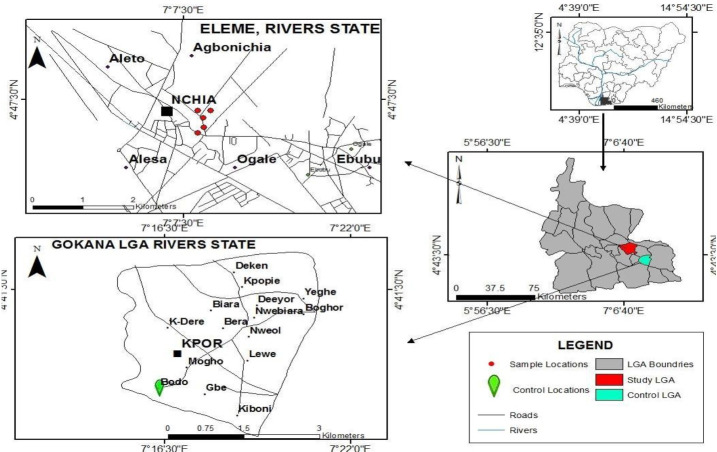
Map of Rivers State, Nigeria, showing the study area and the sampling points in Nsisioken Ogale, Eleme Local Government Area.

**Figure 4. f4-eaht-39-2-e2024015:**
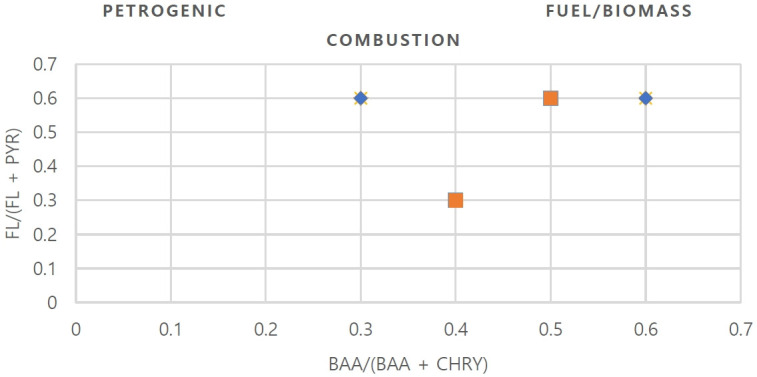
Cross-plot showing diagnostic ratios of groundwater of Nsisioken-Agbi Ogale, Nigeria.

**Table 1.** Sampling sites and characteristics.

No	Sampling sites	Description	Coordinates
1	Well 1	A household well used primarily for bathing, washing, and flushing toilets.	4° 47’ 11.8” N
			7° 7’ 43.3” E
2	Well 2	A household well used for bathing,washing, and flushing toilets.	4° 47’ 2” N
			7° 7’ 39.5” E
3	Well 3	A household well used for bathing,washing, and flushing toilets.	4° 47’ 16.1” N
			7° 7’ 42.8” E
4	Borehole 1	Borehole located near a landfill usedonly for bathing, laundry, andotherdomestic purposes.	4° 47’ 10.8” N
			7° 7’ 44.9” E
5	Borehole 2	Borehole serves as a source of drinking water for residents.	4° 47’ 21.9” N
			7° 7’ 39.5” E
6	Borehole 3	Borehole is an intervention waterproject of the Rivers State governmentthat provides drinking water toresidents. But water isavailableoccasionally.	4° 47’ 15.3” N
			7° 7’ 34.9” E

W1=Well 1, W2 = Well 2, W3 = Well 3, BH1 = Borehole 1, BH2 = Borehole 2, BH3 = Borehole 3.

**Table 2.** Physico-chemical properties of water samples from household wells in Nsisioken-Agbi Ogale in Rivers State, Nigeria and reference limits.

Sampling stations	W1	W2	W3	BH1	BH2	BH3	Reference limits	References
PH	8.20	6.88	6.41	6.62	6.55	6.54	6.5-8.5	[41]
Temperature (℃)	29.6	29.3	29.3	30	30	29.9	Ambient	n/a
DissolvedOxygen (mg/L)	3.27	3.64	3.83	4.11	4.28	5.38	> 5	[42]
EC (µScm^-1^)	500	385.5	310.9	32.78	12.84	11. 21	< 1000	[43]
Total Dissolved Solids (mg/L)	330.9	252.1	200.9	20.42	7.95	6.09	500	[43]
Turbidity (NTU)	9.3	7.8	5.6	5.1	2.9	2.7	< 5	n/a
Sulphate (mg/L)	0.741	0.119	0.246	0.031	< 0.001	0.118	250	[43]
Nitrate (mg/L)	0.240	0.242	0.242	0.247	0.248	0.231	50	[44]
Phosphate (mg/L)	0.080	0.095	0.027	0.105	0.041	0.052	5	-
TPH (µg/L)	5038	2335.8	1662.5	13751.1	7409.2	ND	300 - 500	[45]

**Table 3.** PAHs concentration in groundwater (μg/L) of Nsisioken-Agbi Ogale from 5 sampling stations.

PAHs	Conc. (μg/L) Mean ± S. D					
	W1	W2	W3	BH1	BH2	BH3
Naphthalene	ND	ND	ND	ND	ND	ND
Acenaphthylene	ND	ND	ND	ND	ND	ND
Acenaphthene	ND	ND	ND	ND	ND	ND
Fluorene	ND	ND	ND	ND	ND	ND
Anthracene	ND	ND	ND	ND	ND	ND
Phenanthrene	ND	ND	ND	ND	ND	ND
Fluoranthene	2.0 ± 0.00	2.0 ± 0.0	2.5 ± 0.71	1.5 ± 0.71	0.5 ± 0.71	ND
Pyrene	1.5 ± 0.71	1.5 ± 0.71	2.5 ± 0.71	1.0 ± 0.0	0.5 ± 0.71	ND
Benz[a]anthracene	1.0 ± 1.41	2.5 ± 0.71	1.5 ± 0.71	0.5 ± 0.71	ND	ND
Chrysene	2.5 ± 0.71	2.0 ± 0.0	2.0 ± 0.0	1.0 ± 1.41	ND	ND
Benzo(b)fluoranthene	ND	ND	0.5 ± 0.71	ND	ND	ND
Benzo(k)fluoranthene	ND	ND	ND	ND	ND	ND
Benzo(e)fluoranthene	ND	ND	ND	ND	ND	ND
Dibenzo(a,h)anthracene	ND	ND	ND	ND	ND	ND
Indeno [1,2,3, c,d] pyrene	ND	ND	ND	ND	ND	ND
Benzo [g,h,i] pyrene	ND	ND	ND	ND	ND	ND
∑grand mean PAHs (µg/L)	7.0 ± 2.89	8.0 ± 1.42	9.0 ± 2.84	4.0 ± 2.83	1.0 ± 1.42	-

**Table 4.** Guidelines for PAH diagnostic ratios considered for identification of PAH sources in Nsisioken-Agbi household water samples.

PAH Diagnostic ratio	Value	Source	Reference
FLA / (FLA + PYR)	< 0.4	Petrogenic	[47]
	0.4 - 0.5	Fuel combustion Coal grass and wood combustion	
	> 0.5		
BaA / (BaA + CHRY)	< 0.2	Petrogenic	[48]^1^
	0.2 - 0.35	Fuel Combustion	
	> 0.35	Coal grass and wood combustion	

**Table 5.** PAH diagnostic ratio value from the study.

Diagonistic ratio	W1	W2	W3	BH1	BH2
Fla / Fla + Pyr	0.6	0.6	0.5	0.6	0.5
BaA / (BaA + Chry)	0.3	0.6	0.4	0.3	-

## Supplementary Material

Add short descriptions of supplementary material. This material is available online at www.eaht.org.
