# Supplementary material for: Polycyclic aromatic hydrocarbon in community drinking water, Nsisioken, Nigeria: Source and health risk assessment
Source: Environ Anal Health Toxicol. 2024 Jun 7;39(2):e2024015. doi: 10.5620/eaht.2024015 (PMC11294663; doi:10.5620/eaht.2024015)
Supplement: Supplementary file 1 [file eaht-39-2-e2024015-Supplementary-Tables.pdf]

EAHT
Environmental Analysis Health and Toxicology
eISSN: 2233-6567
OpenAccess
Vol: 39(2), Article ID: e2024015
https://doi.org/10.5620/eaht.2024015

Supplementary Material

Table 6. Hazard Quotients (HQs) of PAHs in the water samples by ingestion.

Non-Carcinogenic PAHs	Ref Dose	KP	W 1		W 2		W 3		BH 1		BH 2	
			Children	Adults	Children	Adults	Children	Adults	Children	Adults	Children	Adults
Fluoranthene	0.04	0.36	3.3	1.3	3.3	1.3	4.0	1.8	1.5	0.8	2.3	1.0
Pyrene	0.03	0.33	3.0	1.3	3.0	1.3	5.3	2.3	2.0	1.0	2.0	1.0
HQ = Σ HI			6.3	2.6	6.3	2.6	9.3	4.1	3.5	1.8	4.7	2.0

Table 7. Hazard Quotients (HQs) of PAHs in the water samples by dermal contact

Non-Carcinogenic PAHs	Ref Dose	KP	W 1		W 2		W 3		BH 1		BH 2	
			Children	Adults	Children	Adults	Children	Adults	Children	Adults	Children	Adults
Fluoranthene	1.24×10^{-2}	0.36	7.3	4.0	2.3	4.0	9.7	4.8	4.0	1,6	5.6	3.2
Pyrene	9.30×10^{-3}	0.33	7.5	3.2	7.5	3.2	11.8	6.5	4.2	2.2	4.3	2.2
HQ = Σ HI			14.8	7.2	9.8	7.2	21.5	9.0	8.2	3.8	9.9	5.4

Copyright © 2024 The Korean Society of Environmental Health and Toxicology/Korea Society for Environmental Analysis This is an Open Access article distributed under the terms of the Creative Commons Attribution Non-Commercial License (https://creativecommons.org/ licenses/by-nc/4.0/) which permits unrestricted non-commercial use, distribution, and reproduction in any medium, provided the original work is properly cited.

Table 8. Potential carcinogenic risks for adults and children in well and borehole water from ingestion

Carcinogenic PAHs	CSF	TEF	KP	ILCR							
				W 1		W 2		W 3		BH 1	
				Children	Adults	Children	Adults	Children	Adults	Children	Adults
Chrysene	0.073	0.01	0.81	7.44×10^{-3}	1.85×10^{-6}	8.0×10^{-7}	1.48×10^{-6}	8.0×10^{-7}	1.48×10^{-6}	8.0×10^{-7}	1.48×10^{-6}
Benz(a) anthracene	0.73	0.1	0.81	8.03×10^{-4}	1.46×10^{-3}	1.0×10^{-3}	1.83×10^{-3}	6.0×10^{-4}	1.11×10^{-3}	4.0×10^{-4}	7.4×10^{-4}
Benzo(b)fluoranthene	0.73	0.1	1.2	ND	ND	ND	ND	4.0×10^{-4}	7.37×10^{-4}	ND	ND
RI = Σ ILCR				8.24×10^{-3}	1.46×10^{-3}	1.0×10^{-3}	1.83×10^{-3}	1.0×10^{-3}	1.85×10^{-4}	4.0×10^{-4}	7.4×10^{-4}

Table 9. Potential carcinogenic risks for adults and children in well and borehole water from dermal contact

Carcinogenic PAHs	CSF	TEF	KP	ILCR							
				W 1		W 2		W 3		BH 1	
				Children	Adults	Children	Adults	Children	Adults	Children	Adults
Chrysene	0.073	0.01	0.81	1.68×10^{-6}	3.69×10^{-6}	1.36×10^{-6}	2.95×10^{-6}	1.36×10^{-6}	2.95×10^{-6}	1.36×10^{-6}	2.95×10^{-6}
Benz(a) anthracene	0.73	0.1	0.81	1.31×10^{-3}	2.92×10^{-3}	1.7×10^{-3}	3.69×10^{-3}	1.02×10^{-3}	2.95×10^{-6}	6.81×10^{-4}	1.48×10^{-3}
Benzo(b)fluoranthene	0.73	0.1	1.2	ND	ND	ND	ND	1×10^{-3}	2.18×10^{-3}	ND	ND
RI = Σ ILCR				1.31×10^{-3}	2.92×10^{-3}	1.7×10^{-3}	3.69×10^{-3}	2.02×10^{-3}	4.4×10^{-3}	6.81×10^{-4}	1.48×10^{-3}
